# The evolutionary origins of the vertebrate olfactory system

**DOI:** 10.1098/rsob.200330

**Published:** 2020-12-23

**Authors:** Guillaume Poncelet, Sebastian M. Shimeld

**Affiliations:** Department of Zoology, University of Oxford, 11a Mansfield Road, Oxford OX1 3SZ, UK

**Keywords:** evolutionary, vertebrates, olfactory, tunicate, lamprey

## Abstract

Vertebrates develop an olfactory system that detects odorants and pheromones through their interaction with specialized cell surface receptors on olfactory sensory neurons. During development, the olfactory system forms from the olfactory placodes, specialized areas of the anterior ectoderm that share cellular and molecular properties with placodes involved in the development of other cranial senses. The early-diverging chordate lineages amphioxus, tunicates, lampreys and hagfishes give insight into how this system evolved. Here, we review olfactory system development and cell types in these lineages alongside chemosensory receptor gene evolution, integrating these data into a description of how the vertebrate olfactory system evolved. Some olfactory system cell types predate the vertebrates, as do some of the mechanisms specifying placodes, and it is likely these two were already connected in the common ancestor of vertebrates and tunicates. In stem vertebrates, this evolved into an organ system integrating additional tissues and morphogenetic processes defining distinct olfactory and adenohypophyseal components, followed by splitting of the ancestral placode to produce the characteristic paired olfactory organs of most modern vertebrates.

## 1. Introduction: olfaction and chemosensation

Olfaction is a form of chemosensation. It is colloquially equated to the sense of smell, the specific sensing of chemicals in the air via the nose and the relaying of this information to the brain via olfactory nerves. However, the precise evolutionary and developmental delineation of the olfactory system becomes blurry when one considers the details. Many vertebrates have a related chemosensory system in the vomeronasal organ, which shares a developmental origin with the main olfactory system but has generally been thought to be devoted to chemical communication between conspecifics. In aquatic vertebrates, such as fish and amphibians, a homologous olfactory system to that of terrestrial vertebrates detects waterborne rather than airborne chemicals, while insects possess a well-described system in their antennae that senses airborne chemicals and is usually called an olfactory system, but is convergently evolved at the system level. Furthermore, the development of the vertebrate olfactory system includes the formation of cells associated with other functions, including that of the pituitary, and there are many vertebrate chemosensory cells that relay information to the brain but that are not part of olfactory systems in the conventional sense. Taste is an obvious example.

Sensing chemicals on the outer side of the cell membrane is a fundamental feature of all cells and sensing environmental chemicals has obvious adaptive advantages. It is therefore not surprising that a diversity of chemosensory mechanisms and systems have evolved in animals. We will not attempt to cover this diversity here, but will focus specifically on the evolution of the vertebrate olfactory system. We will combine two levels of comparison: first, the types of neural cells that develop in the olfactory system (both chemosensory and neurosecretory cell types). Second, the mechanisms that control the specification of olfactory cells and organs. Since vertebrate olfactory cells develop from an ectodermal placode that shares a developmental and evolutionary history with other such placodes, we will also consider placode development and evolution more broadly.

**Figure 1.**
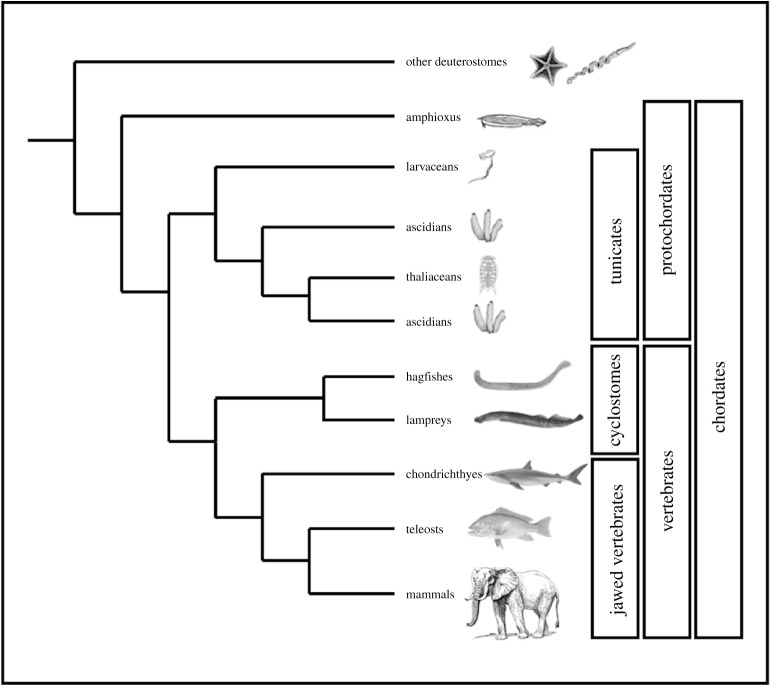
A phylogeny of the chordates showing the relationships of the major lineages discussed in this review. Note that the ascidians appear twice in the tree as they are paraphyletic.

We first summarize what is known about this in jawed vertebrates (also known as gnathostomes). We then compare this to olfactory systems and related cells and structures in other chordates (figure 1): the jawless vertebrates (represented by lampreys and hagfishes, collectively the cyclostomes), the tunicates (including sea squirts and their allies) and the cephalochordates (represented by amphioxus). We will finish with a model for how the olfactory system in living vertebrates evolved.

## 2. The olfactory systems of jawed vertebrates and their neural cell-type derivatives

**Figure 2.**
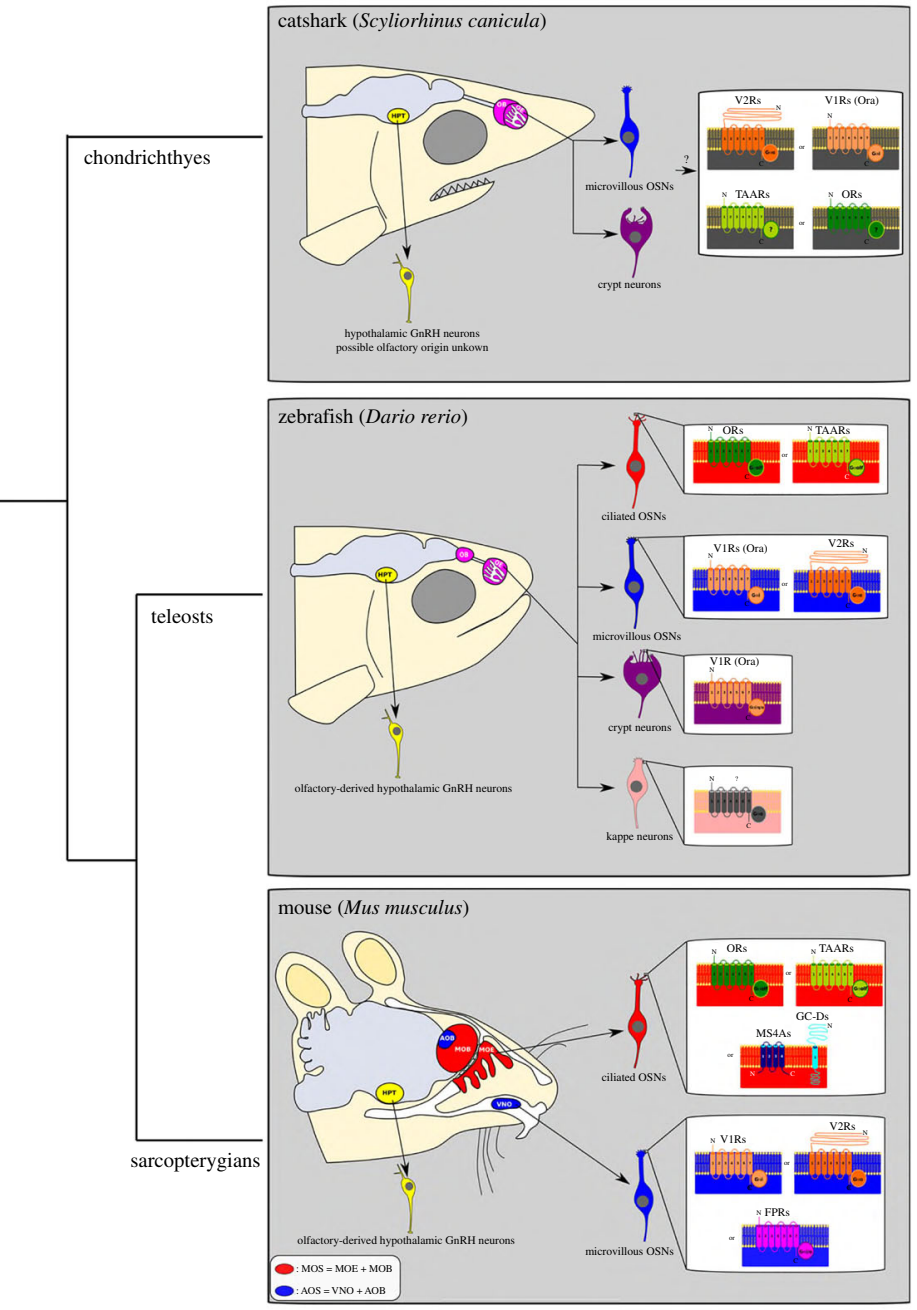
Schematic diagrams of adult organization of the main and accessory olfactory systems in shark, teleost and mammal lineages, including some of the neural cell types discussed in the text and shown in table 1. Note that olfactory-derived GnRH neurons of the terminal nerve and NPY neurons are not represented here. AOB, accessory olfactory bulb; GC-D, guanylate cyclase D; FPR, formyl peptide receptor; GnRH, gonadotropin-releasing hormone; HPT, hypothalamus; MOB, main olfactory bulb; MOE, main olfactory epithelium; OR, olfactory receptor; TAAR, trace amine-associated receptor family; V1R, vomeronasal type 1; V2R, vomeronasal type 2; VNO: vomeronasal organ.

There are two major olfactory subsystems in jawed vertebrates: the main olfactory system (MOS) and the accessory olfactory system (AOS) (figure 2), and relevant neuronal cell types are summarized in table 1. The MOS is historically said to be important for the detection of odorants and the AOS to mainly sense pheromones. However, the systems may overlap functionally and act synergistically [1]. When a chemical stimulus flows into the MOS or AOS, it is detected by olfactory sensory neurons (OSNs) through specific membrane chemoreceptors. Nearly all these chemoreceptors are coupled to a specific G protein subunit *α*, encoded by genes of the GNAL and GNAS families [2]. The different G*α* proteins mediate signal transduction pathways that open cyclic nucleotide‐gated (CNG) ion channels in the MOS or transient receptor potential (TRP) channels in the AOS. These channels trigger a calcium influx in the olfactory neuron cytosol, promoting the opening of calcium-gated chloride channels. The combined effect of calcium and chloride efflux triggers OSN depolarization [3]. OSNs expressing the same chemoreceptor gene send their axon projections via the olfactory nerve to a specific glomerulus in the olfactory bulb. The OSNs of the MOS transmit the chemosensory signal through the main olfactory bulb, which then connects to higher brain centres for the processing of a behavioural response. The OSNs of the AOS target their axons to the accessory olfactory bulb in the rostral telencephalon, which then projects towards the amygdala and hypothalamus, which are involved in aggression and mating behaviours [4].

**Table 1.** Summary of neural cell types developing from the olfactory placode and its derivatives. Abbreviations: MOS, main olfactory system; AOS, accessory olfactory system; FPR, formyl peptide receptor; GC-D, guanylate cyclase D; GnRH, gonadotropin-releasing hormone; OSN, olfactory sensory neuron; OR, olfactory receptor; TAAR, trace amine-associated receptor; MS4A, membrane-spanning 4A receptor; V1R, vomeronasal type 1 receptor; V2R, vomeronasal type 2 receptor.

taxonomic group	location/embryo origin	sensory cell types and receptors	G protein used	additional comments
sarcopterygians	MOS	ciliated OSNs expressing ORs	G*α*olf	
		ciliated OSNs expressing TAARs	G*α*olf	
		ciliated OSNs expressing GC-D family receptors and MS4As family receptors	not G protein coupled	demonstrated only in rodents
	AOS	microvillous OSNs expressing V1Rs	G*α*i	AOS lost in some tetrapod lineages
		microvillous OSNs expressing V2Rs	G*α*o	
		microvillous OSNs expressing FPRs family	G*α*i/o	demonstrated only in rodents
teleosts	single epithelium	ciliated OSNs expressing ORs	G*α*olf	
		ciliated OSNs expressing TAARs	G*α*olf	
		microvillous OSNs expressing V1Rs	G*α*i	
		microvillous OSNs expressing V2Rs	G*α*q	
		crypt cells with cilia and microvilli expressing V1Rs	G*α*i/o/q	
		cap cells, receptor unknown	G*α*o	
chondrichthyes	AOS only	microvillous OSNs likely expressing V2Rs	G*α*o	dominance of V2Rs, with few TAARs, V1Rs and ORs genes. It is not precisely known which receptor is expressed by each sensory cell type
		crypt cells with cilia and microvilli likely expressing V1Rs	unknown	
possibly all gnathostomes	olfactory placode	neuropeptide Y neurons	N/A	migrate from placode to hypothalamus, but this has only been demonstrated in chicken
		GnRH1 neurons	N/A	migrate from olfactory placode to hypothalamus and pre-optic area. It has been demonstrated in osteichthyes but not to date in chondrichthyes. In some species, they also migrate to form the terminal nerve if GnRH3 is absent (functional compensation of paralogue)
		GnRH3 neurons	N/A	migrate from olfactory placode to form the terminal nerve. In some species, they also migrate to the hypothalamus and pre-optic area if GnRH1 is absent (functional compensation of paralogue)

The MOS includes the main olfactory epithelium, which is composed of typical ciliated OSNs. The cilia of the OSNs harbour seven-transmembrane domain G-protein-coupled receptors from the olfactory receptor family (OR), or receptors of the trace amine-associated receptor family (TAAR), on their surface [5]. The ORs are the largest gene family in vertebrates and there may be more than a thousand different genes in the genomes of some species [6]. The TAARs and ORs are coupled to G protein subunit G*α*olf. In the recesses of the mammalian main olfactory epithelium, there is an expression of chemoreceptors from the guanylate cyclase D receptor family, and the MS4A gene family, in the cilia of a specific group of OSNs known as the necklace OSNs. These latter receptor families are not coupled to specific G proteins [7].

The AOS is also known as the vomeronasal system in tetrapods and includes a distinct sensory epithelium containing OSNs bearing microvilli instead of cilia. Like ciliated MOS OSNs, these microvillous OSNs express seven-transmembrane domains G-protein-coupled receptors, but in the AOS, these are chemoreceptors of the vomeronasal type 1 (V1R) or type 2 (V2R) families. V1Rs and V2Rs are associated with the G protein subunits G*α*i and G*α*o, respectively. In rodents, some microvillous sensory neurons of the vomeronasal organ also express members of the formyl peptide receptor family associated with the identification of pathogens and infections [8]. The description of an accessory olfactory system in lungfish suggests that all sarcopterygians primitively had such a dual system [9]; however, the vomeronasal system is not preserved in all tetrapod groups and is absent or vestigial in some lineages like archosaurs (birds and crocodilians) and higher primates [1]. While molecular studies have yet to extend across the diversity of tetrapods, there is a long history of histological and ultrastructural studies of tetrapod olfactory and vomeronasal systems covering many different species. There is insufficient space to detail these studies here and they have been well-reviewed by Eisthen [10]. It is important to note, however, that the tetrapod group may harbour system diversity beyond the simple division of ciliated OSNs in the MOS and microvillous OSNs in the AOS familiar from mammals. For example, both types of OSN are found in the MOS of some urodele amphibians and ciliated OSNs in some lizard and bird species may have their cilia surrounded by microvilli [10].

In teleost fishes, there is no proper vomeronasal organ but a single olfactory epithelium showing morphological features of both the main and accessory systems and with intermingled ciliated and microvillous OSNs [11]. Teleost ciliated OSNs express the ORs and TAARs associated with G*α*olf, similar to the main olfactory epithelium of tetrapods. Similarly, the teleost microvillous OSNs express V1Rs and V2Rs associated with G*α*i and G*α*o, as seen in the vomeronasal organ of mammals. It was also demonstrated in zebrafish that microvillous OSNs form a neural circuit via the dorsomedial olfactory bulb and intermediate ventral telencephalic nucleus (the putative teleost medial amygdala) to the tuberal hypothalamus, similar to the vomeronasal circuit of tetrapods [12]. Teleosts have a third class of OSNs, the crypt cells, which possess microvilli and cilia and express V1R-type (ORA) receptors [2,13]. In zebrafish, there is also a fourth type of OSN, the cap (kappe) cell, whose receptor type is unidentified but known to associate with G*α*o [14]. In Chondrichthyes, it was observed that the sense of smell relies primarily on microvillous OSNs coupled to G*α*o [15]. The chemosensory receptor repertoire of cartilaginous fishes is dominated by the expanded V2R family, although there are also a few OR, TAAR and V1R genes [16]. As in teleosts, there is also the presence of crypt neuron-like cells but their exact receptor is unknown [17]. This peculiar feature and the absence of ciliated OSNs suggest that the cartilaginous fish olfactory system is just an accessory system [18].

Some neurosecretory cells also delaminate from the olfactory placode of gnathostomes. The most well-known are the gonadotropin-releasing hormone (GnRH) neurons involved in the reproductive axis [19,20]. There are three distinct populations of GnRH neurons in the brain of gnathostomes expressing one of the three existing *GnRH* genes: *GnRH1*, *GnRH2* and *GnRH3*. Some species have lost some of these paralogues, but most vertebrates express at least two. Neurons expressing either *GnRH1* or *GnRH2* have been identified in most gnathostomes, while *GnRH3*-expressing neurons are fish-specific [21,22]. The prevailing view is that the GnRH1 neurons and GnRH3 neurons originate from the olfactory placodes, while the GnRH2 neurons are of non-placodal origin and develop within the central nervous system, mainly the midbrain [23,24]. The GnRH1 neurons are key regulators of fertility as an essential part of the hypothalamic–pituitary–gonadal axis (HPG). These cells migrate along axons of the terminal nerve**/**olfactory pathways up to the forebrain where they settle inside the pre-optic and hypothalamic areas [25]. Once settled within the hypothalamus, GnRH1 neurons send their axons to the median eminence and secrete GnRH1 into the portal vessels, where it travels to the adenohypophysis [26]. Here, GnRH1 activates specific receptors of the gonadotrope cells, which then release two crucial hormones for sexual maturation and reproduction, luteinizing hormone and follicle-stimulating hormone [27]. In chick, it was shown that the neuropeptide Y (NPY) neurons also derive from the olfactory placodes and migrate to the hypothalamus along with GnRH1 neurons. NPY neurons control the secretion of GnRH1 by acting directly on GnRH1 neurons [28]**.**

GnRH3 neurons migrate from the olfactory placodes and become components of the terminal nerve whose processes extend anteriorly to the nasal cavity and posteriorly to various brain regions, mediating chemosensory processing and reproduction [29]. The GnRH3 neurons of the terminal nerve (TN-GnRH3) have neuromodulatory effects on the OSNs [30,31]. In addition, the TN-GnRH3 neurons of zebrafish have been demonstrated to be chemosensory, detecting CO_2_ in order to avoid incoming predators [32]. This latter finding suggests that olfactory-derived neurons with dual GnRH/chemosensory abilities could exist in vertebrates (as is advocated for the aATENs of ascidians, see §9 below for details) [33]. In species that lack either *GnRH1* or *GnRH3*, the remaining gene compensates functionally for the lost paralogue by being expressed by the relevant cells. For example in zebrafish, where *GnRH1* is lost, the *GnRH3* gene is expressed in the pre-optic area and hypothalamus (the *GnRH1* territory) in addition of the terminal nerve (the *GnRH3* territory).

It needs to be made clear that the olfactory epithelia of the MOS and AOS also contain non-neural cells that surround the OSNs, such as supporting cells and basal cells. The supporting cells provide physical and metabolic support to the olfactory epithelium. The basal cells are stem cells used to constantly replenish the olfactory epithelium as they can differentiate into either OSNs or supporting cells. The MOS also contains the mucus-producing olfactory (Bowman's) glands whose proteinaceous secretion allows solubilization of odorants in the nasal cavity [25,34]. These cells are important for vertebrate olfactory system function but will not be the focus of this review as they are not easily compared between vertebrates and other chordates.

## 3. Development of the jawed vertebrate olfactory system from the olfactory placodes

The vertebrate cranial placodes are transient ectodermal thickenings of embryonic head and contribute to the developing cranial sensory systems. In jawed vertebrates, the olfactory system develops from a pair of cranial placodes, the olfactory placodes. Other cranial placodes such as the lens, vestibuloacoustic, trigeminal, epibranchial and lateral line placodes contribute to other cranial senses, including sight, hearing, balance, somatosensation, gustation and internal physiological monitoring: their respective cell types and functions have been extensively reviewed elsewhere and will not be further considered here [35–38]. However, one other placode develops in intimate association with the olfactory placodes and warrants further mention. The adenohypophyseal placode forms between the paired olfactory placodes, in front of the extreme anterior of the neural plate. During subsequent development, it becomes internalized through the mouth [39], eventually forming the adenohypophysis and thus having a direct functional connection with olfactory placode-derived GnRH neurons in the hypothalamus.

**Figure 3.**
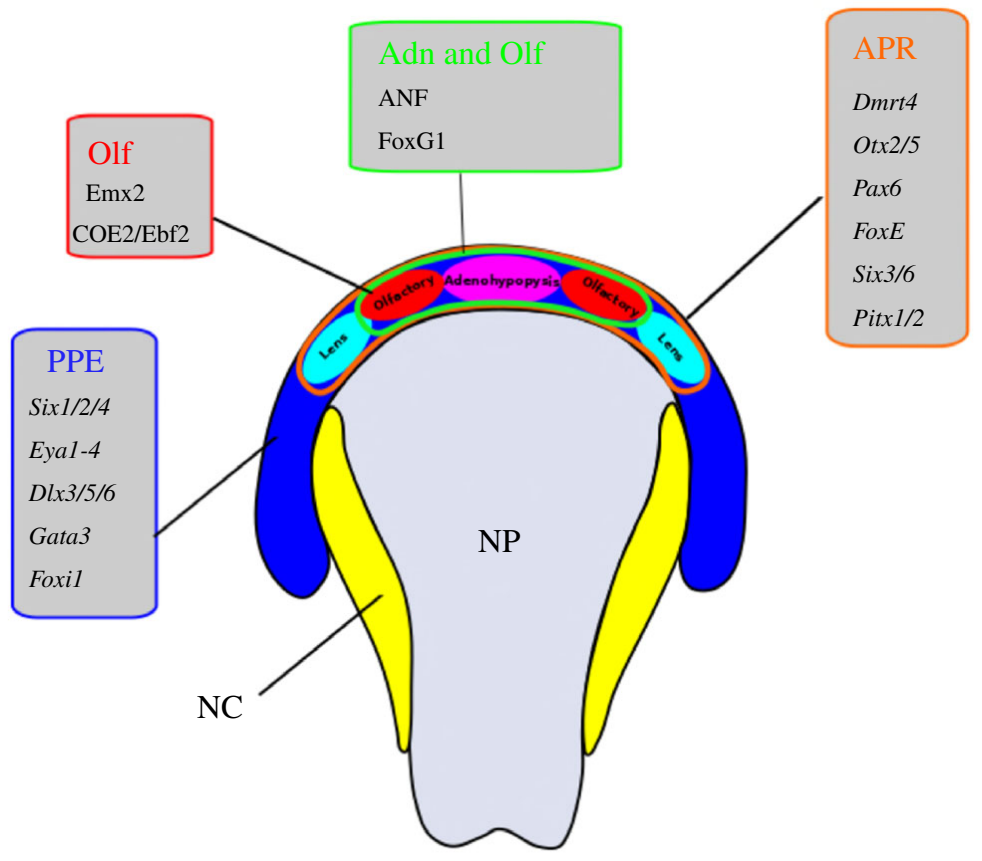
Anterior ectodermal patterning and origin of individual anterior placodes from the pre-placodal ectoderm (PPE) in jawed vertebrates. The PPE is specified by FGF signalling, BMP antagonism and Wnt-antagonism, all coming from the underlying mesoderm. These induce transcription factors within the PPE (blue), which specify precursor regions for multiplacodal areas (coloured outlines) and individual placodes (coloured ovals). Known transcription factors for each are shown (for a more detailed discussion of these genes and genes marking other placodes see [38]). Abbreviations: Adn, adenohypophyseal placode; APR, anterior placodal region; NC, neural crest; NP, neural plate; Olf, olfactory placode.

All the cranial placodes arise from the pre-placodal ectoderm (PPE), a U-shaped cell field around the edge of the anterior neural plate (figure 3). The PPE is specified by fibroblast growth factor (FGF) signalling and bone morphogenetic protein (BMP)- and Wingless-related integration site (Wnt)-antagonism [40]. It is characterized by the expression of pre-placodal competence factors such as the transcription factors (TFs) *Six1/2, Six4/5, Eya1-4, Dlx3/5/6, Gata3* and *Foxi1* [41]. During the development, the PPE subdivides in specific regions along the anteroposterior axis to give rise to individual cranial placodes. In particular, the lens, adenohypophysis and olfactory placodes are defined anteriorly through the combinatorial expression of TFs such as *Dmrt4, Otx2/5, Pax6, FoxE, Six3/6* and *Pitx1/2* [42]. BMP signalling promotes specification of an olfactory fate, while extended BMP exposure time promotes lens fate [43]. FGF signalling from the anterior neural plate is known to block expression of the lens marker gene *Pax6* and to promote the expression of *Dmrt4*, an olfactory placodal gene [44,45]. Olfactory placodes are further characterized by the expression of the transcription factor genes *Emx2* and *COE2* (*Ebf2*), among others [39].

In addition to the ectodermal cells from the PPE, the olfactory placodes become associated with migratory neural crest cells [25,35]. These give rise to the olfactory ensheathing cells, which are glial cells that envelop the bundles of olfactory axons [34,46,47]. The possible contribution of neural crest to other cell types in the olfactory placode such as the GnRH neurons is debated and controversial, and there is no coherent vision on the lineage origin of the major neural cell types associated with the olfactory sensory epithelia of vertebrates [20,46,48]. Precise lineage cell-tracing data in zebrafish argue against a contribution from the neural crest and support the view that all the different neuronal populations within the olfactory epithelium originate from overlapping pools of progenitors in the PPE [19]. However, a recent analysis of GnRH1 neurons in the mouse olfactory placode argued for a heterogeneous origin, with neural crest and PPE-derived GnRH1 neurons [49]. It is possible these apparently conflicting reports reflect genuine differences between species.

## 4. The olfactory system in jawless vertebrates

There are only two surviving lineages of jawless vertebrates, the lampreys and the hagfishes (figure 1), though fossils show a much wider diversity of extinct lineages [50]. There are many similarities in head development between jawed and jawless vertebrates, including sensory systems and the central nervous system. For example, most jawed vertebrate placodes are clearly identifiable in lampreys [51,52]; cranial nerve organization is similar [51] and gross brain organization well-conserved [53,54]. There is, however, an important difference in the olfactory system. Jawed vertebrates have paired nostrils leading to paired olfactory sacs and derived from the paired olfactory placodes. Lampreys and hagfish have a single median nostril, a condition known as monorhiny. This develops to form a single anterior medial placode known as the nasohypophyseal placode, which combines characters of both olfactory and adenohypophyseal placodes and produces a single, medial nasal (olfactory) sac.

Despite this difference, both lampreys and hagfishes have well-developed olfaction. Adult sea lamprey uses odours from conspecific larvae (including dihydroxylated tetrahydrofuran fatty acids and some bile acids) to select the best streams for spawning based on their larval population [55–59]. Hagfishes are usually found in deep water and their olfactory organ seems particularly efficient as they have been shown to be among the first fish species to locate chemical signals of decaying prey [60,61]. Electrophysiological recordings indicate that their olfactory sensory neurons are particularly sensitive to amino acids [62]. In addition to the conventional olfactory system, hagfishes have specialized chemosensory structures named ‘Schreiner organs’ all over the body epidermis [63]. Their ecological role is not known, though we can speculate they may help with directional chemosensation in the absence of paired olfactory membranes.

## 5. The olfactory system of lamprey and its neural cell-type derivatives

**Figure 4.**
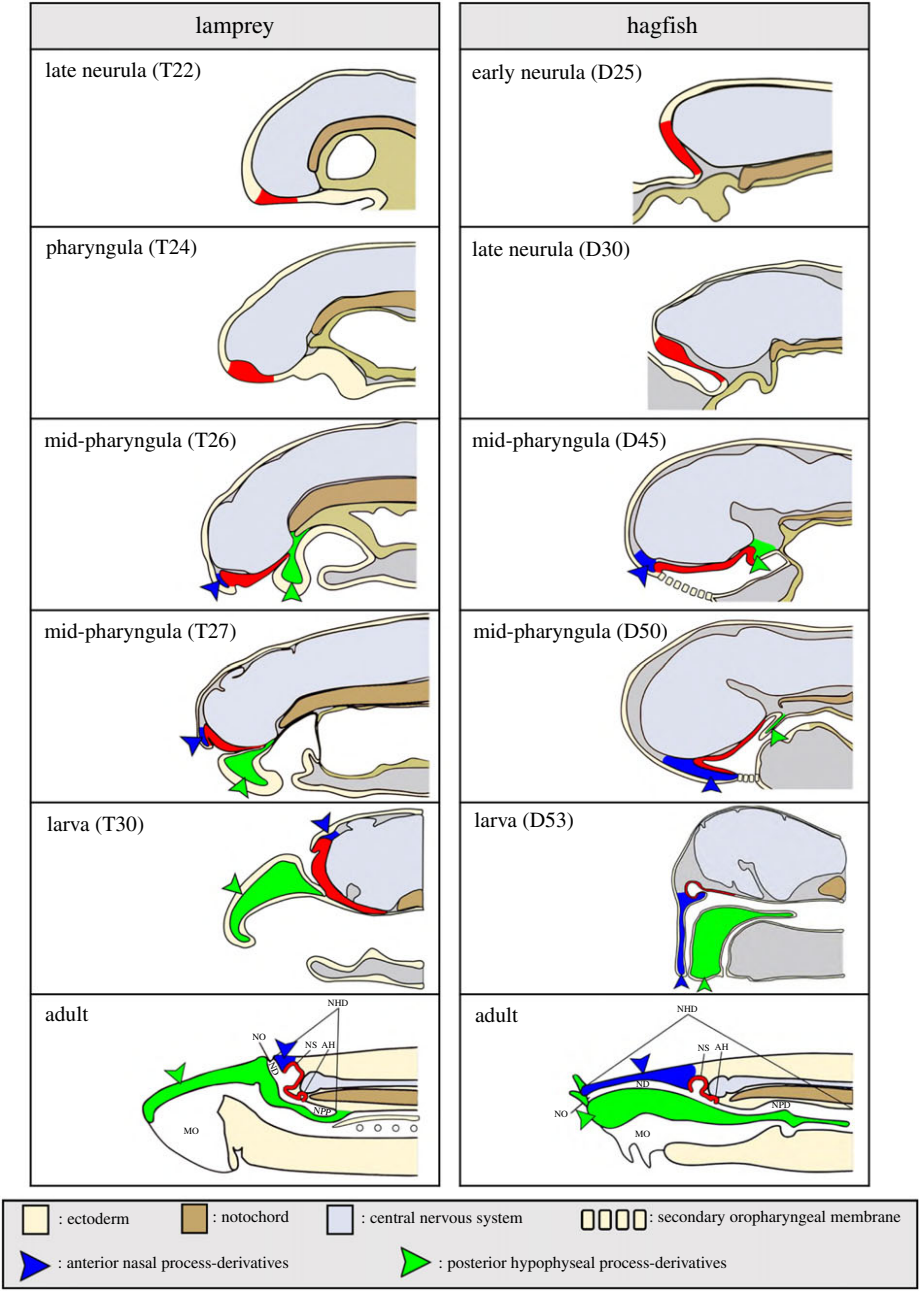
Comparative development of nasohypophyseal placode (NHP) in lampreys and hagfishes. The NHP is labelled in red. At the mid-pharyngula stage, when development is the most similar in cyclostomes, the NHP is rostrocaudally bordered by ventral growth of two ectomesenchymal processes, the anterior nasal process (blue) and the posterior hypophyseal process (green). Abbreviations: AH, adenohypophysis; MO, mouth; ND, nasal duct; NHD, nasohypophyseal duct; NO, nostril; NPD, nasopharyngeal duct; NPP, nasopharyngeal pouch; NS, nasal sac; Figure adapted from [64] with permission. T and D denote lamprey and hagfish embryo staging, respectively [65,66].

In larval and adult lamprey, the single olfactory organ is composed of three elements: the nasal duct, the nasal sac and the nasopharyngeal pouch (figure 4) [67]. The nasal tube opens externally as a single nostril on the dorsal head surface. In adult lampreys, the nasal tube contains a valve that serves to introduce and expel water into the entrance of the nasal sac, the chemosensory part of the organ [68]. When larval lamprey metamorphos into adults, the olfactory organ extends and changes from an epithelial lined tube to a nasal sac with lamellar folds [69]. In the sea lamprey *Petromyzon marinus*, the nasal sac wall is divided into 25 folds [68]. Each fold is lined with the main olfactory epithelium, which is mainly composed of tall, narrow, ciliated OSNs [70,71]. However, OSNs in the main olfactory epithelium display at least three distinct morphotypes, with some not necessarily ciliated but with microvillar-like protrusions. These different OSNs have been proposed to be similar to the ciliated OSNs, microvillous OSNs and crypt cells found in teleost fishes [72]. The OSNs in the main olfactory epithelium express the three chemoreceptor gene families identified in the sea lamprey genome, the ORs, TAARs and V1Rs; the V2R gene family seems to be gnathostome-specific as it is apparently absent in lamprey [73–76]. The OSNs of the main olfactory epithelium send projections mainly to the non-medial region of the olfactory bulbs, but also send some projections to its medial region [77]. In the caudoventral portion of the peripheral olfactory organ, there is an accessory olfactory organ [78], which is covered with the accessory olfactory epithelium containing short, rounded, ciliated neurons [79,80]. The accessory olfactory epithelium sends projections exclusively to the medial olfactory bulb, which connects to the posterior tuberculum creating a motor response from olfactory inputs [80,81].

It has been hypothesized based on anatomical and molecular evidence that the lamprey accessory olfactory epithelium, coupled with the dorsomedial telencephalic neuropil, is the putative homologue of the tetrapod vomeronasal system [82,83]. However, it remains questionable whether the so-called ‘main’ and ‘accessory’ olfactory epithelia of lamprey are indeed homologous to the main and accessory olfactory epithelia of sarcopterygians. Characters that point to homology are that the lamprey main and accessory olfactory epithelia have differences in their respective pathways and that distinct G protein subtypes are used for signal transduction, with G*α*olf located only in the OSNs projecting to the non-medial olfactory bulb [84]. Hence, different types of G proteins are used from those of OSNs projecting to the medial glomeruli, a similarity shared with the vomeronasal organ. However, there is a notable difference in that ORs, TAARs and V1Rs are not differently expressed between the lamprey olfactory epithelia as opposed to the tetrapod vomeronasal organ and main olfactory epithelium [80,82]. This difference could represent an intermediate and ancestral condition before the exclusive shift to vomeronasal receptor genes, as seen in the AOS of gnathostomes.

In lampreys, current evidence suggest that GnRH neurons of the pre-optic area and hypothalamus are not derived from the nasohypophyseal (NHP) placode, contrary to what is observed in jawed vertebrates [85,86]. Immunohistochemical investigation concluded that lamprey GnRH neurons were never seen in association with the NHP placode during embryonic development, and it was hence hypothesized that GnRH neurons originate within proliferative zones of the diencephalon in developing lamprey, not in the olfactory system [86]. However, other data supporting this difference to jawed vertebrates are lacking.

## 6. The olfactory system of hagfish and its neural cell-type derivatives

As in lampreys, the olfactory system of adult hagfish is composed of three main parts: a nasal duct, a nasal sac and a nasopharyngeal duct (figure 4) [87,88]. Hagfishes also possess a single nostril just above the mouth, surrounded by two nasal tentacles on each side and by a dorso-median lip. The nasal duct leads to the nasal sac anterior to the brain [88,89]. A valve is present in an oblique position inside the nasal duct and serves to manage water flow in the duct towards the nasal sac [90]. The latter receives a continuous flow of water during respiration as the water flows from the nostril and is ejected through the gill openings as, unlike in lamprey, the nasopharyngeal duct does not end blindly but opens into the pharynx [88,89]. The nasal sac is divided into seven olfactory laminae and the olfactory epithelium is composed of two types of OSNs, ciliated or microvillous. In adult hagfishes, GnRH neurons have been identified in the diencephalon [91–93]. However, the embryonic development of these cells has not been investigated and nothing is known about the potential association or shared origin of hagfish GnRH neurons with the olfactory system.

## 7. Olfactory system development in jawless vertebrates

The developmental trajectories of lamprey and hagfish systems are shown in figure 4. Early in development the single median nasohypophyseal placode is characterized by orthologous molecular markers to those seen in the gnathostome olfactory placode. The entire nasohypophyseal placode territory expresses *Six3/6A* and *Soxb1* in hagfish [64] and *Pax6* in lamprey [94,95], consistent with the expression of the gnathostome orthologues *Pax6*, *Six3/6* and *Sox2/3* in both the olfactory and adenohypophyseal placodes [37]. At the late neurula stage of lamprey and hagfish embryos, the anterior part of the nasohypophyseal placode becomes the likely olfactory territory as it expresses *FGF8/17/18*, the orthologue of *FGF8* in gnathostomes. Reciprocally, the posterior part becomes the adenohypophyseal territory as it shows *PitxA* expression [64,96]. At the pharyngula stage in lamprey, the nasohypophyseal placode forms a thickened area of the ventral ectoderm, anterior to the mouth cavity. Morphogenesis of this region is coordinated with that of two ectomesenchymal processes (figure 4) and has been described elsewhere [64]. Important points to note are that the nasohypophyseal placode extends an epithelial cell process posteriorly to establish close contact with the definitive hypothalamic region, thus forming a pituitary similar to that of jawed vertebrates in combining central nervous system and placode-derived parts. The anterior part of the nasohypophyseal placode, which will form the future olfactory epithelium, is characterized by the expression of olfactory developmental gene markers such as *OtxA, CoeA, CoeB, EmxA* and *EmxB* [95–99].

As lamprey embryos approach the larval stage, the anterior part of the nasohypophyseal placode differentiates as the nasal sac, composed of a thick columnar epithelium and covering the rostral aspect of the telencephalon, whereas the posterior (as the future adenohypophysis and consisting of an epithelium of a few cell layers) extends caudally to the level of the optic chiasma [96]. In late hagfish embryos, the nasohypophyseal duct and oral cavity grow posteriorly relative to the position of the adenohypophysis. The presumptive nasohypophyseal duct is tilted inward in an oblique position unlike that of lamprey, which is situated more vertically. In addition, the posterior end of the nasohypophyseal duct in hagfishes ruptures into the pharynx (figure 4). Thus, the nasohypophyseal duct in hagfishes opens secondarily into the pharynx [64].

To summarize, there are many similarities in development, gene expression and cell-type derivatives between the olfactory systems of jawless and jawed vertebrates. There are also some important differences. Most notably, jawless vertebrate systems develop from a single medial placode combining olfactory and adenohypophyseal progenitors that separate during morphogenesis, and form a single medial olfactory system and not the paired systems of jawed vertebrates. Fossil data suggest monorhiny is the ancestral condition [100–102], so a single medial olfactory/adenohypophyseal placode as seen in living jawless fish is probably also ancestral (though note the description of paired nasal sacs in some vertebrate stem lineage fossils, and that despite monorhiny, lampreys and hagfishes have a pair of olfactory nerves like gnathostomes, meaning that some questions remain over this inference [103–105]). It is also unclear whether GnRH neurons are olfactory placode derivatives in jawless fish like GnRH1 and GnRH3 neurons in gnathostomes, or are born within the central nervous system like gnathostome GnRH2 neurons.

## 8. Potential olfactory system homologues in protochordates

The tunicates and cephalochordates are collectively known by the paraphyletic term ‘protochordates’ (figure 1). They are usually considered not to have cranial placodes in a strict sense [106]. However, they do have some ectodermal patterning mechanisms that appear conserved with those of vertebrate placodes. These have been best studied in the ascidians, which have two areas of ectoderm postulated to be placode homologues: one just anterior to the neural plate and a potential homologue of the olfactory and adenohypophyseal placodes (discussed more below) and the other paired and lateral to a more posterior neural plate. Data on ectodermal patterning have been recently reviewed in detail elsewhere [36,38,107] and the reader is referred here for more discussion on this aspect. Protochordates also have several types of morphologically distinct ectodermal sensory cells. Some have been proposed to be chemosensory, though this is mainly based on cytological features and the expression of marker genes, and no cell advocated as chemosensory has had this experimentally evaluated [107–112].

**Figure 5.**
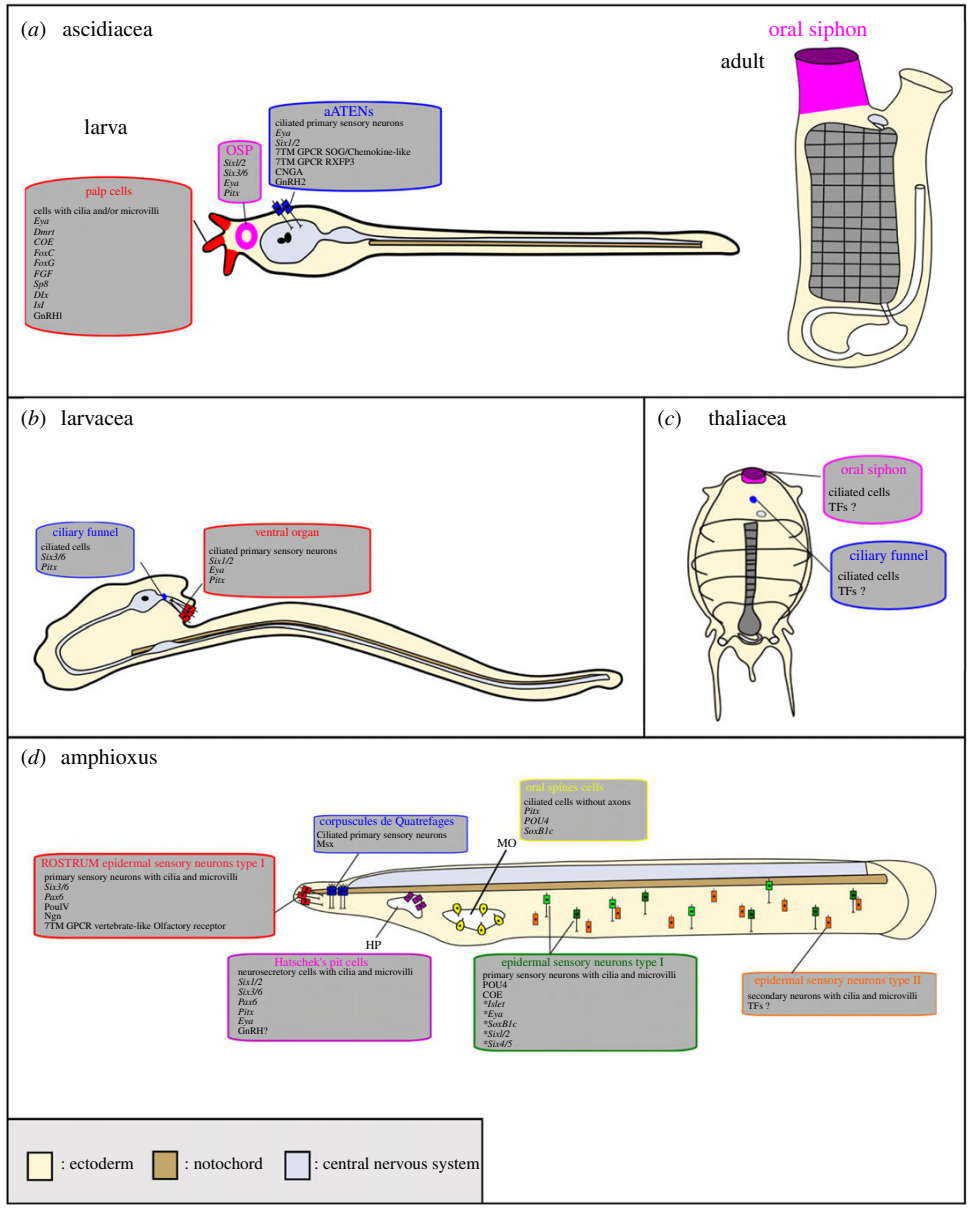
Possible olfactory sensory cells in protochordates and the genes they express. (*a*) Schematic drawing of an ascidian larva and adult of *Ciona intestinalis*. The ascidian swimming larva has three regions suspected to hold or give rise to putative chemosensory cells: the palp cells (red), the oral siphon primordium (OSP, pink) which becomes the oral siphon in adults, and the anterior trunk epidermal neurons (aATENs, blue). (*b*) Schematic drawing of the adult larvacean, *Oikopleura dioica*. Two main regions are believed to host chemosensory cells: the ciliary funnel (blue) and the ventral organ (red). (*c*) Schematic drawing of the adult thaliacean, *Thalia democratica*. Chemosensory cells are thought to be present in two places: the oral siphon (pink) and ciliary funnel (blue). (*d*) Schematic drawing of an amphioxus larva and the cells suspected to be chemosensory. The types of cells and relevant gene expression are indicated. Note that the Type 1 sensory cells are heterogeneous as indicated by different colours. Genes listed with an asterisk are only expressed in subsets of sensory cells. For more detail see [107,114]. Abbreviations: HP, Hatschek's pit. MO, mouth. Panel D adapted from [36] with permission.

Amphioxus species are all quite similar in gross morphology. Tunicates are more disparate. The majority of species fall into the ‘Ascidiacea’, a paraphyletic grouping united by an ascidian type life cycle, with a motile tadpole larva and a sessile adult that may be solitary, or may form colonies by asexual reproduction [113]. Other tunicates are motile as adults. This includes the larvaceans, which maintain the tadpole body plan throughout life, and thaliaceans (figures 1, 5*a*–*c*).

## 9. Putative olfactory cell homologues in Ascidiacea

Several studies have reported a failure to identify orthologues of the vertebrate olfactory receptor genes in tunicate genomes, and orthologues of chemosensory receptor genes used by insects and nematodes could also not be found [115–117]. Tunicate orthologues of V1R and V2R genes are also missing [74,75,118]. Furthermore, although TAAR-like genes have been proposed to be present in tunicates and amphioxus [75], these authors did not give details and others have disagreed with this as they failed to identify TAAR orthologues [74,119]. It would be surprising if tunicates do not sense any chemical cues, especially with an active swimming larval stage that in most species needs to choose an appropriate settlement site for metamorphosis. Further, in adult tunicates, there is evidence that the oral siphon can sense chemicals as sensitivity to acids, bases, salts and anaesthetics has been shown [120]. It is therefore probable that alternatives to olfactory receptor genes are used in these organisms. Recently, some seven-transmembrane protein encoding genes have been identified and proposed to fulfil the role (discussed more below), though protein function is not established [33,121].

The model ascidian *Ciona intestinalis* is by far the best-studied species in this field. During neurulation, the border region to the neural plate gives rise to several types of sensory cell, two of which are relevant to the discussion of olfactory placode evolution: a subset of anterior trunk epidermal neurons (the aATENs) and the palp sensory cells (PSCs) (figure 5*a*). Both arise from the cells just anterior to the neural plate, in the region mentioned above as a prospective olfactory/adenohypophyseal homologue. The developmental pathway leading to the specification of these sensory cells has been expertly reviewed elsewhere [122], and the reader is referred here for a detailed description of how they form and then acquire distinct identities.

The aATENs are four ciliated primary sensory neurons that in *Ciona* larvae come to be located in the epidermis dorsal to the cavity of the sensory vesicle, their simple equivalent of the brain [123,124]. These neurons express *GnRH*, a cyclic nucleotide-gated channel (*CNGA*) and, as mentioned above, two seven-transmembrane G-protein-coupled receptors: a relaxin-3 receptor (*RXFP3*) and a somatostatin/opioid/galanin/chemokine-like receptor (*SOG/Chemokine-like*) [33]. This led the authors to propose that the aATENs had dual chemosensory and neurosecretory activities, combining functions of vertebrate olfactory-derived OSNs and GnRH neurons in a single cell [33]. However, caveats to this are that functional chemosensory activity has not been experimentally shown, and that the two seven-transmembrane G-protein-coupled receptors identified in these cells are not orthologous to the vertebrate olfactory receptors. Testing sensory cells for chemical stimulation has been an experimental challenge in a developmental system like *Ciona* as the small cells make electrophysiology difficult. The recent development of fluorescent cell activity reporters will probably resolve this technical bottleneck [121,125].

Ascidian tadpoles also have ciliated primary sensory neurons in each of the palps, the anterior adhesive organs by which larvae appear to sense and bond to attachment sites [112,126]. Like aATEN cells, the palps develop from the area anterior to the neural plate postulated to be an olfactory/adenohypophyseal placode homologue and express many regulatory genes that are important for olfactory placode development in vertebrates, like *Eya, COE, Dmrt, FoxC, FoxG, FGF, Sp8, Dlx* and *Isl* [127–130]. It is possible that the palp sensory neurons are involved in tadpole settlement site selection via chemoreception, though again this is not experimentally validated and others have suggested that a different cell type in the palps may be chemosensory [121]. The palps are also known to produce GnRH in cells likely to be neuronal as they seem to possess long axons [131], suggesting an evolutionary link to the olfactory placode-derived GnRH neurons in gnathostomes

In addition to these larval cell types, the oral siphon primordium (OSP; figure 5*a*) develops in the same region, with its progenitors sandwiched between those that give rise to the aATENs and those that give rise to the palps. The primordium maintains the expression of placode marker genes including *Six1/2, Six3/6* and *Pitx* into the larval stage and at metamorphosis forms the oral siphon, which in adults includes sensory cells in many ascidian species. Some of these cells may be chemosensory [120] but their developmental origin has yet to be traced so it is still possible they do not derive from the primordium. A structure called the ciliated funnel opens into the oral siphon and connects to a gland associated with a ganglion, the combination of which is termed the neural complex. This dual structure is reminiscent of the pituitary and homology has been considered [132], with the ciliated funnel sensing water entering the oral siphon and perhaps chemosensory. As for other ascidian sensory cells, this has not been experimentally validated.

## 10. Putative olfactory cells in larvaceans

It has been suggested that the ventral organ (figure 5*b*) of the larvacean tunicate *Oikopleura dioica* is homologous to the vertebrate olfactory organ. The ventral organ possesses about 30 primary sensory cells with cilia that protrude externally into sea water. These neurons are located in an ectodermal slit-like pocket and send their axons to the rostral-most CNS [133]. Furthermore, developmental genes like *Eya, Pitx* and *Six1/2*, which are important in the development of vertebrate olfactory and adenohypophyseal placodes, are expressed in the primordia of the larvacean ventral organ. Therefore, the *Oikopleura* ventral organ placode was said to be homologous to the ectoderm of the ascidian palps based on gene expression and structure [134]. Again, the sensory function of the cells has not been tested.

## 11. Putative olfactory cells in thaliacea

Thaliaceans comprise the pelagic tunicates salps, doliolids and pyrosomes. There has been less research in thaliaceans in comparison to other tunicate classes. In salps and doliolids, the cells located around the oral lips (figure 5*c*) have been suggested to be chemoreceptors based on the observation that salps respond to chemical stimuli positioned in proximity of the oral opening [135]. Another possible chemosensory structure is the ciliated funnel, as discussed above with respect to ascidians and which is present in thaliaceans. In the thaliacean *Thalia democratica*, it has been suggested that the ciliated funnel could possibly collect odorants from the environment. [136]. Developmental and genetic confirmation of this hypothesis is currently lacking, however.

## 12. A summary of olfactory system homology in tunicates

Experiments show tunicates respond to chemical stimuli, and position and developmental genetic data point to the ectoderm just anterior to the neural plate as the homologue of the vertebrate olfactory and adenohypophyseal placodes. This area also produces sensory neurons, at least some of which express GnRH. It remains to be experimentally shown whether cells are chemosensory, and if so what receptors they use considering homologues of vertebrate receptor families are lacking. However, assuming they are chemosensory, the data strongly support the contention that the ancestor of the tunicates and vertebrates had a chemosensory system developing from the ectoderm alongside the anterior neural plate and that has evolved into the systems we see today in living tunicates and vertebrates. It is less clear how complicated this ancestral system was. In tunicate larvae, it consists of scattered sensory cells that may combine multiple functions, rather than a larger organ system with the morphogenesis and specialized cell types of vertebrates. This points to a simple grade of organization in the common ancestor. Adult tunicates have morphologically more complex organs with more cells, which could point to a more complex ancestral state. However, cell functions are again unknown, developmental genetics poorly understood and cell lineages unclear. This makes discriminating between conservation and parallelism or convergence challenging. Until additional data are available, the analysis of outgroups like amphioxus provides the alternative route to inferring ancestral states.

## 13. Putative olfactory cells in amphioxus

Amphioxus are known to exhibit sensitivity to chemicals dissolved in sea water, with most triggering an escape response [137,138]. Ectodermal territories with the gene expression, morphogenetic processes or focused neurogenesis characteristic of vertebrate placodes have not been identified, with the possible exception of Hatschek's pit (see below). Ectodermal sensory cells are present but are broadly scattered throughout the general epidermis (figure 5*d*), and at least some of these cells develop in the ventral ectoderm of the early embryo under BMP signalling [139]. It is not known which sensory modalities are mediated by each cell but some have been suggested to be chemosensory based on cytology and molecular markers (reviewed in [36,107,140,141]). There are two major subtypes of these epidermal sensory cells: type I and type II. The type I sensory cells are primary sensory cells with a long cilium surrounded by microvilli. The type II sensory cells are secondary neurons and have a short cilium encircled by a collar of microvilli [142–144].

The type I cells have been proposed to be mechano- and/or chemoreceptors [145]. The population of type I sensory neurons is molecularly heterogeneous and subsets express orthologues of transcription factor genes seen during vertebrate olfactory placode development such as *COE, Islet, POU4, SoxB1c, Six1/2, Six4/5* and *Eya* [114,146–149]. Some may be chemosensory, for instance, the expression of *COE* by type I sensory cells located caudally along the flanks of the amphioxus body might suggest this as COE genes are expressed in the olfactory placodes of vertebrates and chemosensory neurons of the organism such as *Caenorhabditis elegans* [97,150]. In particular, the anterior of amphioxus is interesting as it includes type I ciliated primary sensory neurons coming from an ectodermal region expressing olfactory placodal markers like *Pax6, Six3/6, Ngn* and *POU4* [146,148,151]. Furthermore, in the amphioxus *Branchiostoma floridae* genome, there are more than 50 genes orthologous to the vertebrate ORs [115,117,152,153], and some of these anterior type I cells express at least one of these genes suggesting they may be cell-type homologues of OSNs [154].

Type II cell morphology, with a collar of external projecting microvilli supported by a modified cilium, also suggests chemosensory rather than mechanosensory function. The cells could be homologous to vertebrate primary olfactory sensory cells, which means they would have secondarily lost their axons. They could also be homologous to secondary vertebrate chemosensory cells like the taste buds or solitary chemoreceptor cells. However, the molecular identity of the cells is unknown as their late appearance in larval development [143] has so far hindered molecular studies. All these hypotheses also need to be confirmed by physiological studies as none of these cells has had their sensory modality validated.

In addition of type I and type II epidermal sensory cells, there are other specialized sensory cell types in amphioxus that have been inferred to be chemosensory; the cells from the corpuscles de Quatrefages, the oral spine cells and Hatschek's pit cells (figure 5*d*). The corpuscles de Quatrefages are located in the rostrum and form two clusters of specialized ciliated primary sensory neurons that have one to four sensory cells with two cilia each, surrounded by up to seven sheath cells [155,156]. They have been speculated to form a mechanosensory organ but could well be chemosensory [157]. The ciliated oral spine cells around the mouth opening do not possess axons or microvilli, though they do express *Pitx, POU4* and *SoxB1c*, which are vertebrate anterior placodal markers [114,158]. These specialized cells have been suggested to be mechanoreceptors but also proposed to be homologous to vertebrate taste cells [159].

In adult amphioxus, Hatschek's pit is a structure located in the roof of the oral cavity and sends a projection dorsally around the notochord to contact the ventral brain, an organization similar to the relationship between adenohypophysis and hypothalamus of the vertebrate pituitary system [36,160]. The idea that Hatschek's pit and pituitary may be homologous organs is over 100 years old, and molecular analysis has added some support to this since the developmental precursor to Hatschek's pit (known as the pre-oral pit) expresses the vertebrate anterior placode markers *Six1/2, Eya, Pitx, Pax6* and *Six3/6*. There has also been a suggestion that Hatschek's pit includes chemosensory cells because they carry microvilli and cilia, and are exposed to water flowing into the mouth and so are in contact with potential odorants [161]. This hypothesis requires experimental corroboration as the cytoarchitecture of the Hatschek's pit cells suggests that they are neurosecretory, and they do not have axons.

Immunochemistry with antibodies raised to vertebrate GnRH proteins has been used to suggest that GnRH neurosecretory cells are present in the amphioxus neural tube, and possibly also Hatschek's pit [162,163]. Neural tube GnRH cells are candidates for homologues of the olfactory-derived GnRH neurons of vertebrates, but nothing is currently known about their development: they might migrate in from an ectodermal territory like vertebrate GnRH1/3 cells, but also could be born within the central nervous system like vertebrate GnRH2 cells. The expression of GnRH in Hatschek's pit has also been questioned as it could not be detected in a later study, which only identified GnRH immunoreactivity in the central canal of the anterior nerve cord [164]. This difference in the detection of GnRH could be owing to genuine biological causes (for example, a difference in the reproductive state of the animals examined might affect GnRH expression), or might reflect experimental artefacts from fixation or antibody cross reactivity. Additional work is needed to clarify this.

Overall, Hatschek's pit appears similar to the vertebrate adenohypophysis but not the olfactory system. However, even here there is an important difference in that Hatschek's pit derives from the pre-oral pit of the larva, which itself develops from an anterior head cavity, an endodermal derivative. The adenohypophysis is an ectodermal placode derivative. The lineages of the relevant Hatschek's pit cell types through this developmental process need to be confirmed for certainty, but if they too derive from endoderm then homology would imply that the capacity to build this organ and its associated cell types has transferred from one germ layer to another in chordate evolution. Such a shift in cell fate could have occurred through the shifting in the expression domains of the relevant transcription factors, as proposed by Schlosser [37]. This possibility has had recent support from cell lineage studies in zebrafish, which have demonstrated that in this species some adenohypophysis cells may naturally derive from the endoderm during normal development [165]. While this could be a derived character of zebrafish and not reflect the ancestral condition, it irrespectively shows confinement of pituitary cell fates by germ layer is not as strict as historically imagined.

## 14. A summary of olfactory system homology in amphioxus

In general, the scattered ectodermal sensory neurons of amphioxus are poor candidates for olfactory cell homologues. Most are not anteriorly located and while some express genes that mark vertebrate placode cells, there are also substantial differences in their developmental programmes. Most importantly their early induction and regulation are different as they form far from the neural plate in ventral ectoderm under high Bmp signalling [139]. As such they more resemble another type of sensory neuron in tunicates, those in the ventral tail fin [122], and not aATENs, palp cells or vertebrate placode cells, which all originate in the neural plate boundary. Sensory cells in the anterior of amphioxus larvae may be the exception to this owing to their possible expression of amphioxus OR genes and anterior location. However, as yet their developmental genetics, cell lineage and sensory functions have not been determined and these data would be needed to convincingly test a hypothesis of homology. Hatschek's pit remains the best candidate for an adenohypophysis homologue, though this too has counterarguments as discussed above, and the expression of GnRH here remains to be convincingly established.

With respect to inferring the organization of olfactory sensation in the common ancestor of amphioxus and other chordates, the data do not support the presence of morphogenetic processes building an olfactory organ at this stage in evolution. Rather, chemosensation may have been mediated by scattered sensory cells. Amphioxus OR gene expression data suggest assigning some of these as cell-type homologues of vertebrate OSNs but more work on their development is needed to support this. It would be especially important to know what specifies OR-expressing cells and if their lineage traces back to the anterior neural plate border.

## 15. A model for vertebrate olfactory system evolution

**Figure 6.**
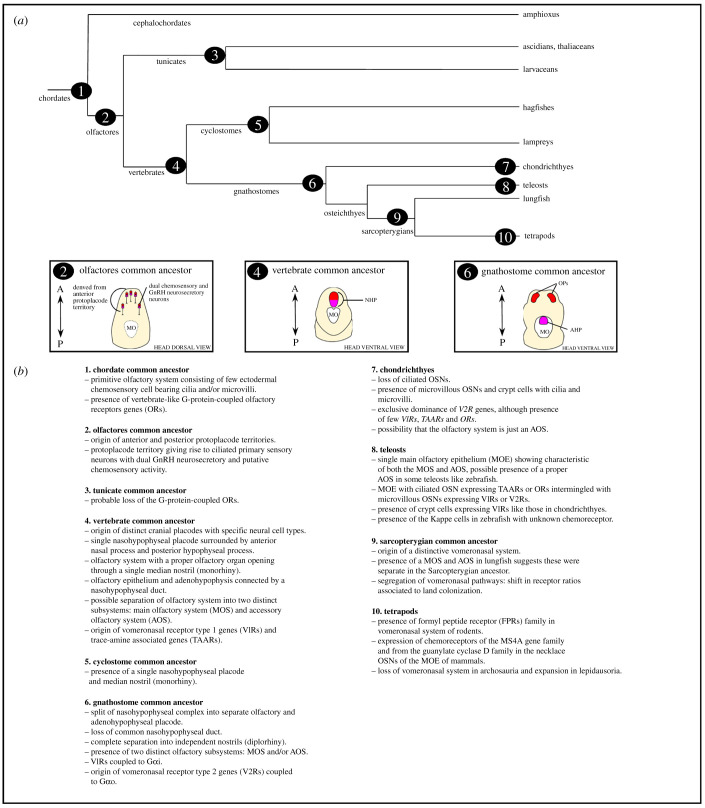
Schematic phylogeny of chordate lineages. Numbers in (*a*) relate to the key in (*b*) and indicate where key events in the evolution of the olfactory system are likely to have occurred. Abbreviations: AHP, adenohypophyseal placode. MO, mouth. NHP, nasohypophyseal placode. OP, olfactory placode.

By plotting genes, developmental processes, cell types and tissues onto a phylogeny of the chordates we arrive at a model for how the olfactory systems of living vertebrates evolved (figure 6). Chemosensation is an ancient sense and was likely mediated by scattered sensory cells in the epidermis of the chordate common ancestor (figure 6–1). Some of these cells probably expressed orthologues of the vertebrate OR genes, as still seen in living amphioxus. By the common ancestor of the tunicates and vertebrates (collectively the Olfactores), specialized ectodermal territories homologous to vertebrate placodes had evolved. We have not discussed the detailed evidence for this here as it has been recently and extensively reviewed [36,38,107]; however, the data suggest the Olfactores ancestor had two placode-like territories and that the anterior of these is the source of the olfactory and adenohypophyseal placodes of vertebrates (figure 6–2). In living tunicates, the anterior placode territory persists and is the source of GnRH and probably chemosensory cells, though the tunicate lineage also lost conventional OR genes.

By the common ancestor of the vertebrates (figure 6–4) a well-defined anterior placode combining olfactory and adenohypophyseal progenitors had evolved, as well as the morphogenetic processes by which it built distinct olfactory and adenohypophyseal systems. It is possible this included distinct main and accessory olfactory system components, including the evolution and deployment of *V1R* receptor genes. We do not yet know whether it also included the progenitors of GnRH neurons that then migrated to the pre-optic area and hypothalamus as this has not been determined in cyclostomes. Key innovations that map to the origin of vertebrates therefore include: (i) the evolution of new types of receptor gene; (ii) as proposed by Schlosser [107] for placode evolution more generally, changes in the control of progenitor proliferation turning single neurons into a neurogenic organ; (iii) mechanisms for specifying subpopulations of OSNs expressing different types of receptor; (iv) changes in morphogenesis including the incorporation of neural crest cells and interactions between placode ectoderm and cranial ectomesenchyme.

In the common ancestor of jawed vertebrates (figure 6–6), this combined placode separated into a medial adenohypophyseal placode and paired olfactory placodes, possibly facilitating the evolution of paired nostrils as seen in all living jawed vertebrates. Subsequently the loss of main or accessory olfactory systems has occurred in some vertebrate lineages, with concomitant shifts in dependence on ORs, V1Rs and V2Rs (figure 6–7, 6–10). It is also interesting that studies of model systems have now identified additional olfactory chemosensory mechanisms beyond these well-known receptors, such as the MS4As, TAARs and FPRs. The evolutionary ancestries of these are less well known and worthy of more study. These findings come from just a handful of species, raising the possibility that the many thousands of less well-studied vertebrate species may harbour additional surprises on this front and that the diversity of olfactory mechanisms deployed by vertebrates may be far higher than currently understood.

## Data accessibility

This article has no additional data.
